# A Class-Imbalanced Deep Learning Fall Detection Algorithm Using Wearable Sensors

**DOI:** 10.3390/s21196511

**Published:** 2021-09-29

**Authors:** Jing Zhang, Jia Li, Weibing Wang

**Affiliations:** 1 School of University of Chinese Academy of Sciences, Beijing 100049, China; zhangjing@ime.ac.cn (J.Z.); wangweibing@ime.ac.cn (W.W.); 2 Institute of Microelectronics of Chinese Academy of Sciences, Beijing 100029, China

**Keywords:** fall detection, class imbalance, deep learning, wearable sensor

## Abstract

Falling represents one of the most serious health risks for elderly people; it may cause irreversible injuries if the individual cannot obtain timely treatment after the fall happens. Therefore, timely and accurate fall detection algorithm research is extremely important. Recently, a number of researchers have focused on fall detection and made many achievements, and most of the relevant algorithm studies are based on ideal class-balanced datasets. However, in real-life applications, the possibilities of Activities of Daily Life (ADL) and fall events are different, so the data collected by wearable sensors suffers from class imbalance. The previously developed algorithms perform poorly on class-imbalanced data. In order to solve this problem, this paper proposes an algorithm that can effectively distinguish falls from a large amount of ADL signals. Compared with the state-of-the-art fall detection algorithms, the proposed method can achieve the highest score in multiple evaluation methods, with a sensitivity of 99.33%, a specificity of 91.86%, an F-Score of 98.44% and an AUC of 98.35%. The results prove that the proposed algorithm is effective on class-imbalanced data and more suitable for real-life application compared to previous works.

## 1. Introduction

Falls have become the second largest threat to the health of people worldwide, and most of the people who die from falls are over 60 years old. The elderly are at the greatest risk of suffering from falls. Falls result in fatal injuries, such as paralysis, hip fracture, head injury, etc. [1]. More than 37.3 million falls occur each year that are severe enough to require medical attention [2]. With the development of society, the aging of the world’s population is of concern. Moreover, the number of elderly people living alone is constantly increasing. In the context of living alone, when an elderly individual falls, it is difficult for them to seek help on their own without a fall detection system (FDS). If the individual does not receive timely medical attention, there may be serious consequences. It is important to rescue elderly individuals in a short time after a fall happens, and the application of FDS has become very significant in this context.

**Figure 1:**
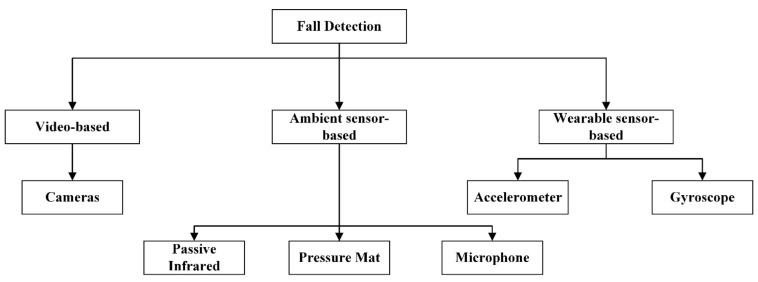
Classification of fall detection system approaches.

Many researchers have focused on human fall detection and have carried out a great deal of work. In the past decade, a large number of fall detection programs have been proposed, which can be divided into the following three categories: video-based [3,4,5,6,7], ambient sensor-based [8,9,10,11] and wearable sensor-based [12,13,14,15,16,17,18], as shown in Figure 1. The sensitivity and the specificity of the video-based methods can reach 97% and 99%, respectively [4], indicating that they can accurately identify the occurrence of falls. However, visualization tools such as cameras used for video image detection are usually fixed, so they are more suitable for indoor use. Moreover, these devices have limitations, such as being easily blocked by objects, and cameras are not privacy-preserving. Furthermore, ambient sensor-based methods are less disruptive to people’s lives, and the sensitivity can reach 97% while the specificity can reach 95% [8]. Nevertheless, ambient sensor-based detection needs to be deployed in advance. In actual applications, there are disadvantages such as high deployment costs and limited detection range. By contrast, wearable device-based detection can solve most of the problems of the above systems. Only part of the movement data is collected, thus resolving the privacy and safety issues, and there is no restriction on the range of motion. Additionally, the sensitivity and the specificity of wearable device-based methods can reach 97.79% and 96.52%, respectively [18]. Even so, wearable devices also suffer from some problems, such as limited battery life, or the wearer may forget to reattach the equipment after bathing, etc. After overall consideration, wearable sensor-based fall detection has become the focus of developers recently.

Even though most of the existing fall detection algorithms are trained and detected on datasets in which the ratio of ADL and fall events is balanced, class imbalance is a serious problem in real applications. When a class imbalance exists within training data, the classifier will typically over-classify the majority group due to its increased prior probability.

In view of the challenge mentioned above, solving such problems is the main motivation of our work. This article focuses on fall detection algorithms on class-imbalanced data, which can effectively extract the characteristics of fall activities from a small number of fall data, and we propose the Class-Imbalanced Deep Learning Fall Detection (CDL-Fall) algorithm. The proposed solution is compared against deep learning algorithms including Convolutional Neural Networks (CNNs), Long Short-Term Memory units (LSTM), DeepSense and a Residual Network (ResNet). The results of the experiment show that the proposed algorithm is effective for class-imbalanced fall data.

The rest of this paper is organized as follows: Section 2 discusses the related works. The details of the proposed method are explained in Section 3. Section 4 shows the experimental settings and results. Section 5 concludes the paper.

## 2. Related Work

In the past decade, a great deal of wearable-based fall detection solutions and class imbalance solutions have been proposed.

### 2.1. Fall Detection

Fall detection algorithms can be divided into three categories: threshold-based, machine learning-based and deep learning-based methods.

#### 2.1.1. Threshold

The threshold method is the earliest proposed algorithm for fall detection. The body will impact the ground when falls happen. Consequently, the value of the accelerometer will suddenly increase during this impact. The threshold method is to set one or more thresholds to detect this impact.

Bourke et al. [13] calculated the root-sum-of-squares of the three signals from a tri-axial accelerometer. The upper and lower fall thresholds were set in order to separate fall events from ADL. Casilari et al. [19] assumed that there were two consecutive stages: free fall and impact. The method identified a fall occurrence if the value of the signal magnitude vector exceeded the lower and upper thresholds during a preset observation time window. Li et al. [20] proposed a three-phrase fall detection process. The acceleration amplitude for stationary postures was less than 0.40 g and the rotational rate amplitude for stationary postures was lower than 60°/S. The threshold of the angle between the trunk and the gravitational vector was set at 35°.

The threshold method offers ease of calculation and implementation in a wearable-based system, but the shortcomings are obvious: false alarms often occur for fall-like activities (such as sudden running), and the robustness of the algorithm is poor.

#### 2.1.2. Machine Learning

In order to improve the robustness of the algorithms, machine learning algorithms have been applied to fall detection. Machine learning can establish a mathematical model based on some features in the data, and it can process large amounts of data.

Giuffrida et al. [21] selected the 10 most frequently leveraged features in different combinations of features to train the parameters of a Support Vector Machine (SVM) model. Yu et al. [22] proposed a fall detection algorithm based on the Hidden Markov Model (HMM). This model did not rely on the subjectivity of feature engineering and manual segmentation, and the raw acceleration signals were processed by Gaussian distributions for hidden states. In the article by Martinez-Villaseor et al. [23], four well-known machine learning classifiers were used for comparison: Random Forest (RF), Support Vector Machine (SVM), Multilayer perception (MLP) and K-Nearest Neighbors (KNN).

Machine learning algorithms can effectively distinguish falls from fall-like activities. However, the limitation is that the data characteristics need to be preset. Selecting fall-related features from a large number of features is a challenge for this algorithm.

#### 2.1.3. Deep Learning

In recent years, deep learning algorithms have been widely used in computer vision, image processing, text generation, speech recognition and other problems. Due to their powerful feature extraction ability, deep learning algorithms have also played a great role in the field of fall detection.

Wang et al. [24] proposed a Cascade and Parallel Multi-State Fall Detection (CMFALL) algorithm that combined a threshold algorithm and Convolutional Neural Network (CNN) algorithm. The method based on a self-attention Convolution Neural Network was lightweight and accurate. Additionally, Long Short-Term Memory (LSTM) is the most popular and frequently used structure in sequence models. As described by Waheed et al. [25], the Noise-Tolerant Fault Detection System (NT-FDS) algorithm used Bidirectional Long Short-Term Memory (BiLSTM) to distinguish falls from ADL in the presence of missing values in data.

Deep learning algorithms can automatically extract features from sensor signals and do not need prior knowledge. Compared with other algorithms, the deficiency is that a large amount of data are needed to train the model and the computing cost is high. However, for more accurate detection, a deep learning model is the best choice.

### 2.2. Imbalance Algorithms

In the above-mentioned fall detection studies, most of them used class-balanced datasets for feature extraction and model parameter training (Table 1). However, fall detection is a class-imbalanced scenario of detecting a problem, where the majority of the activities are healthy. The imbalance ratio is the ratio between the number of samples in the majority class and the number of samples in the minority class, which is defined as follows:

(1) $$\rho =\frac{number\;of\;samples\;in\;majority\;class\;}{number\;of\;samples\;in\;minority\;class}$$

Methods to solve the class imbalance in machine learning can be divided into two categories: data-level methods and algorithm-level methods.

#### 2.2.1. Data-Level Methods

Data-level algorithms mainly include over-sampling and under-sampling. In binary classification problems, over-sampling is the duplication or generation of samples from the minority group. In the Synthetic Minority Over-Sampling Technique (SMOTE) algorithm [26], new samples are generated along the line between the minority examples and their selected nearest neighbors. Lee et al. [27] proposed a two-phase learning method that combined under-sampling with transfer learning to solve the high imbalance problem. This method pre-trained a deep CNN by randomly under-sampling the majority group, and performed fine-tuning using all data.

Data-level methods modify the training distributions in order to decrease the level of imbalance. The algorithms are suitable for scenarios where there is little difference in the amount of data in each class, and they are highly dependent on data.

#### 2.2.2. Algorithm-Level Methods

Related research to solve the problem of class imbalance at the algorithm level is mainly to optimize the loss function: Lin et al. [28] reshaped the standard cross-entropy loss such that it down-weights the loss assigned to well-classified examples. Another modifying loss function method was developed by Wang et al. [29]. The proposed method defined the mean false error (MFE) and mean squared false error (MSFE) to capture the errors from both the majority class and minority class equally. Fuqua et al. [30] developed a cost-sensitive classification scheme within a deep convolution network for the imbalance problem. Moreover, Buda et al. [31] experimented with adjusting the CNN output threshold on a dataset preprocessed by the SMOTE algorithm. The output threshold divided the outputs of the network into each class by the prior probabilities.

In brief, the methods are modified to take a class penalty or weight into consideration, or the decision threshold is shifted in a way that reduces the bias towards the majority class.

## 3. Proposed Method

Although previous approaches have advanced the performance of FDS, they could not perform well on class-imbalanced datasets. The aim of this work is to distinguish a few fall events from a great deal of ADL and improve the robustness of the algorithm. The adopted method is based on a deep learning approach, and is optimized for class-imbalanced wearable sensor data.

### 3.1. Residual Learning

In this research, fall detection is a binary decision task (0 for ADL and 1 for Falls). There are already many DL models used to solve such classification tasks, including CNN and RNN models. The CNN architecture can extract the frequency domain features from images successfully. RNN models are usually used when dealing with long sequential data, such as sentences. Since the data are segmented in the preprocessing stage, the frequency domain characteristics are more important than the temporal features. The proposed algorithm is based on the CNN structure.

**Figure 2:**
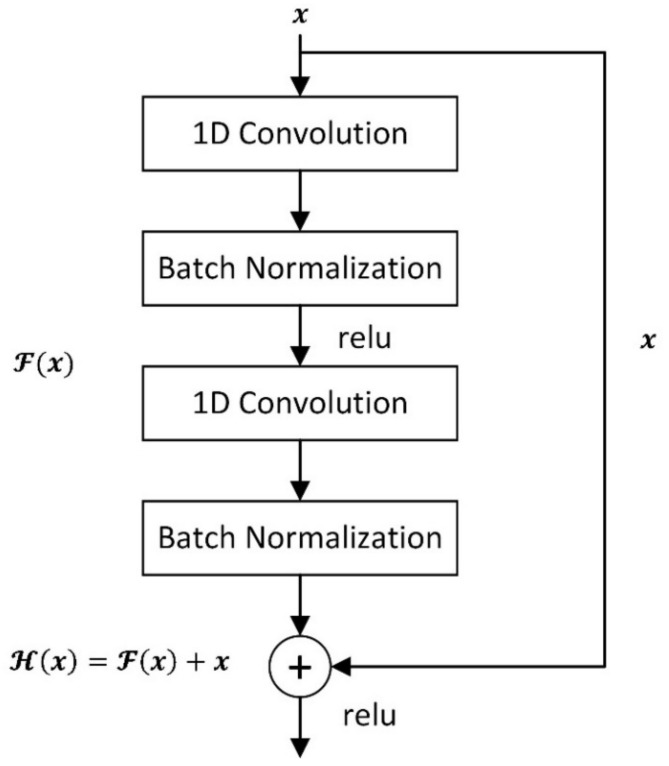
The residual learning block.

The Residual Neural Network (ResNet) [32] applies residual learning to construct the model. Residual learning could settle the problem of vanishing/exploding gradients, and residual learning is defined as Equation (2):

(2) $$𝓗\left(x\right)=𝓕\left(x\right)+x,$$

where 𝓗 represents the output of the two-layer residual learning block. This function is performed by a shortcut connection and element-wise addition. Furthermore, the output of stacked layers can asymptotically approximate the residual function as Equation (3):

(3) $$y=𝓕\left(x,\left\{W_{i}\right\}\right)+x,$$

where x and y are the input and output vectors of the layers considered. The $W_{i}$ is the weight in the weight matrix and the residual learning block is shown in Figure 2. The function $𝓕\left(x,\left\{W_{i}\right\}\right)$ represents the residual mapping to be learned.

### 3.2. Output Threshold Moving

The train dataset can be represented as $\left\{\left(X_{1},y_{1}\right),\;\left(X_{2},y_{2}\right),\;\left(X_{3},y_{3}\right)\ldots \left(X_{M},y_{M}\right)\right\}$, where $X_{i}$ represents the feature vector of the data and $y_{i}$ represents the class of the data. The train dataset is used to train the classifier, which gives an estimate of the posterior probability $P(y^{\prime }=1|X^{\prime })$ for any instance $X^{\prime }$. The class of $X^{\prime }$ is determined by assessing whether the class probability exceeds a pre-defined threshold λ. The sum of possibility is 1. The samples with possibility $P\left(y^{\prime }=1|X^{\prime }\right)\;\geq \;\lambda$ are classified as 1, and the other samples are classified as 0. In class balance tasks, the threshold is set to 0.5 by default because all samples have the same contribution to the model training. For class-imbalanced problems, the classifier may be biased with respect to the majority class; output threshold moving is a simple approach that causes the prediction result of the classifier to focus on the minority group and assign more instances to the minor class. Furthermore, this method is easy to implement on the already trained model.

We define f as the frequency of the minority samples in the train dataset. The classifier is used to predict the test dataset $\left\{\left(X_{1}^{'},y_{1}^{'}\right),\;\left(X_{2}^{'},y_{2}^{'}\right),\;\left(X_{3}^{'},y_{3}^{'}\right)\ldots \left(X_{N}^{'},y_{N}^{'}\right)\right\}$, and the estimated probability of the minority group is defined as ${\hat{P}}_{i}=P\left(y_{i}^{'}=1|X_{i}^{'}\right).$ The $f_{\lambda }$ is defined as the frequency of the estimate minority samples when given the threshold λ. Under this condition, the estimate minority samples are those whose ${\hat{P}}_{i}=P\left(y_{i}^{'}=1|X_{i}^{'}\right)\;\geq \;\lambda$. The formula that is used to determine the threshold is

(4) $$\hat{\lambda }=argmin_{\lambda }\left|f-f_{\lambda }\right|,$$

where $\hat{\lambda }$ is used as the threshold that matches the class distribution of the predictions to that of the train dataset.

**Figure 3:**
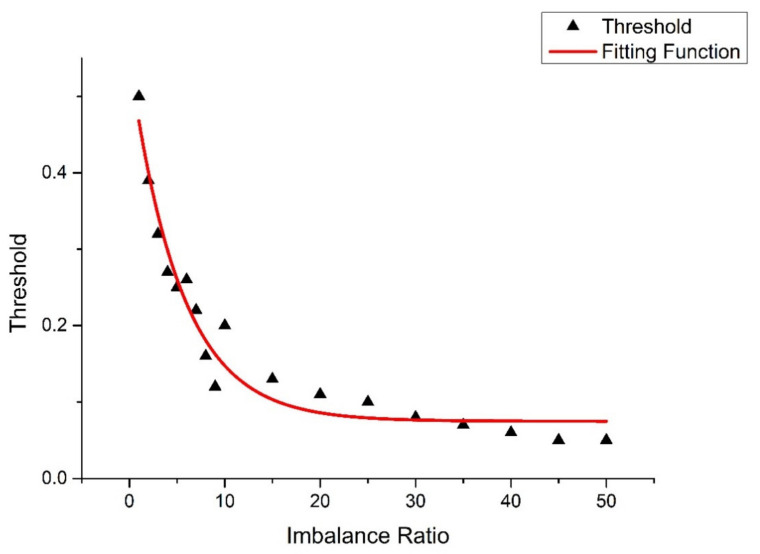
The fitting function between the imbalance ratio and the threshold.

In the experiment, it was noticed that the threshold is related to the imbalance ratio of the dataset. The higher the imbalance ratio of the dataset, the more biased the threshold towards the minority class. In order to determine the relationship between the two, the preprocessed data are randomly selected to form a dataset with different imbalance ratios, and the thresholds under different imbalance ratios are determined based on the above method. The relationship between the imbalance ratio and threshold is fitted according to the experimental results as follows:

(5) λ*=k×e−ρ10×k+k10

where ρ is the imbalance ratio and *k* is the default threshold, usually 0.5. Figure 3 shows the fitting function. The output threshold moving method uses $\lambda^{*}$ to redefine the output decision threshold when classifying samples.

**Figure 4:**
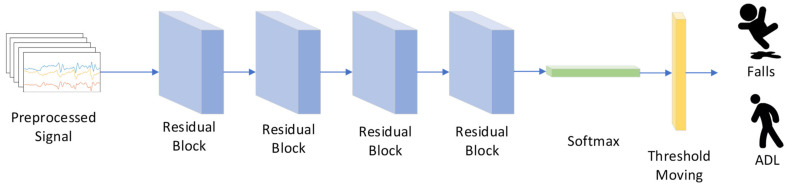
The structure of CDL-Fall network.

The proposed method adjusts the output threshold of ResNet. Figure 4 shows the network structure.

Before they are input into the network, the initial data need to be preprocessed. The details of the preprocessing method are given in Section 4.2. The input signals are passed to four residual blocks; each residual block contains two convolution layers, two batch normalization layers and two rectified linear units. The convolution layers transform the input signals into feature maps using 1×3 kernels as in Equation (6):

(6) $$X_{k}^{i,j}=\sigma \left(b_{j}+\overset{n}{\underset{a=1}{\sum }}w_{a}^{j}x_{k+a-1}^{0,j}\right)$$

where $X_{k}^{i,j}$ is the value of *j* feature map in layer *i*, and $b_{j}$ is the bias of *j* feature map.$\;w_{a}^{j}$ denotes the weight for *j* feature map. σ is the rectified linear unit (ReLU) activation function (Equation (7)):

(7) $$\sigma =max\left(0,x\right)$$

The batch normalization layer performs the normalization for each training mini-batch, and it can use higher learning rates and be less careful about initialization [33]. In order to obtain the possibility of each class, the feature maps are fed into the fully connected layer with a softmax activation function, defined as Equation (8):

(8) $$f\left(x_{j}\right)=\frac{e^{x_{j}}}{\sum_{j=0}^{n}e^{x_{j}}}$$

where $x_{j}$ is the *j* input of softmax. Most classification models convert the probability into prediction results using a default threshold of 0.5. In the CDL-Fall network, the threshold moving layer adjusts the classification threshold based on the imbalance ratio. After being trained with a large amount of data, the CDL-Fall network can correctly identify the fall signal from the class-imbalanced data.

## 4. Experimental Design and Results

### 4.1. Dataset and Labeling

The algorithm proposed in this research needs to be trained and validated on a large and reliable dataset. There are already many publicly available wearable sensor datasets, which are shown in Table 2.

**Figure 5:**
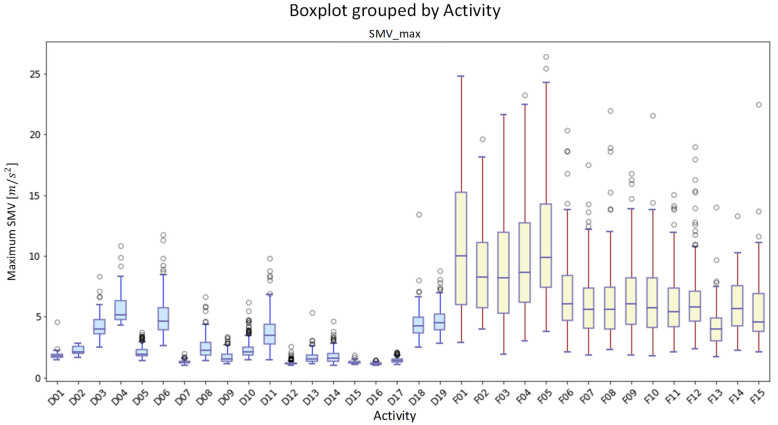
The boxplot of maximum of signal magnitude vector ($SMV_{max}$) of the acceleration. The rectangle represents the distribution range of data and the discrete points represent a few abnormal data.

After comparison, the SisFall dataset [42] is found to be the most suitable for this experiment. The SisFall dataset is generated with the collaboration of 38 volunteers (23 young adults and 15 elderly people). Moreover, there are 19 types of ADL events and 15 types of fall events in the dataset. The data are collected by two accelerometers and a gyroscope, which are worn on the waists of participants. In order to collect all the features of activities, the sensors are operated with the original frequency sample of 200 Hz. Figure 5 shows the distribution of the maximum of signal magnitude vector ($SMV_{max}$) values for all data in each type of event, and the *SMV* can be computed as:

(9) $$SMV_{i}=\sqrt{{\left|A_{xi}\right|}^{2}+{\left|A_{yi}\right|}^{2}+{\left|A_{zi}\right|}^{2}}\;\;m/s^{2}$$

where $A_{xi}$, $A_{yi}$ and $A_{zi}$ define the three components of the acceleration vector for the *i*-th sample in the direction of the *x*, *y* and *z*-axes, respectively. The maximum of the *SMV* ($SMV_{max}$) is defined as:

(10) $$SMV_{max}=max\left\{SMV_{i}\;:i\;\in \;\left[1,N\right]\right\}$$

where *N* indicates the length (number of samples) of each event. As shown in Figure 5, the data distribution of ADL and fall events in the SisFall dataset is stable and clearly differentiated. Therefore, the SisFall dataset is selected as the data source in this experiment.

### 4.2. Data Preprocessing

Initial data from the dataset need to be labeled and segmented before they can be used for model training and validation.

**Figure 6:**
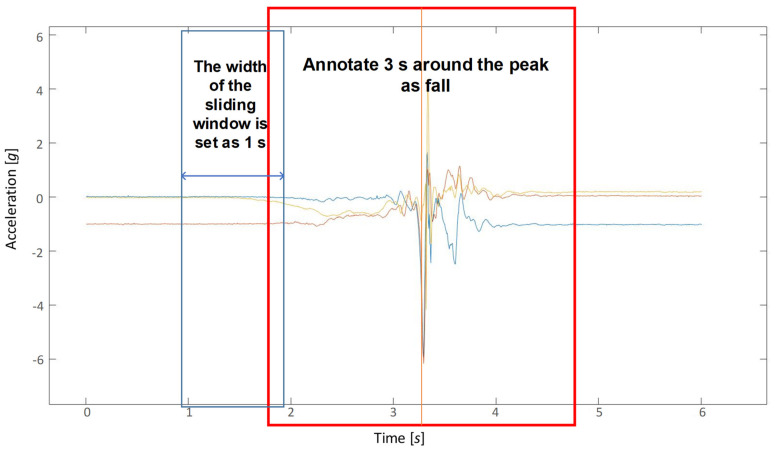
Sliding window strategy for data augmentation.

In initial fall event files, there is a long period of activity before the actual fall data. Sampling is performed at 3 s around the peak values in initial fall event files and they are labeled as falls. Then, all data are augmented by using a sliding window strategy in order to obtain the samples, and the width of the sliding window is set as 1s. The segmentation of the original data and the method of sliding window are shown in Figure 6. The preprocessed dataset includes 94,786 ADL events and 8439 fall events. In consequence, the imbalance ratio is 11.23 and the threshold of experiment $\lambda^{*}$ is set as 0.1.

### 4.3. Evaluation Method

**Figure 7:**
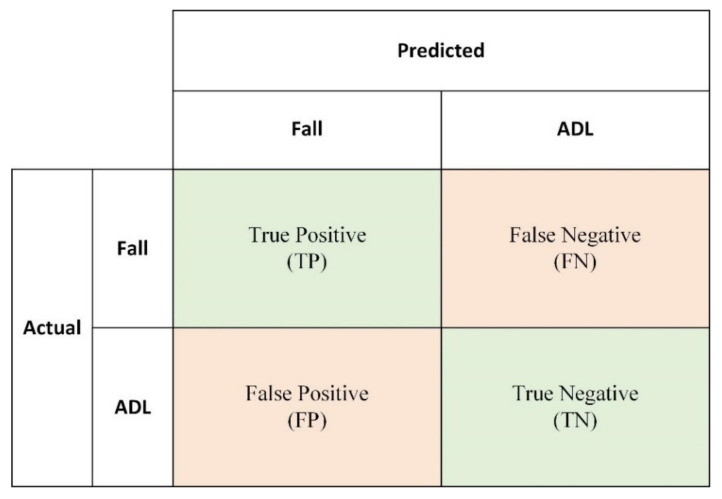
The confusion matrix of fall detection.

There have been many methods to evaluate the performance of different classifiers. All methods are based on the four outcomes of the classifiers, which are elements of the confusion matrix (Figure 7). The confusion matrix is shown as follows:

- True positive (TP): The ADL events have been correctly classified.
- True negative (TN): The fall events have been correctly detected.
- False positive (FP): Fall events that have not been detected.
- False negative (FN): A false alarm situation occurs.

The most widely used method to evaluate the performance of a classifier is accuracy. Accuracy (Equation (11)) is a proportion that can reflect the overall correctly classified samples. However, it has some limitations; for example, accuracy is easily affected by abnormal big data. In a situation in which the training data are class-imbalanced, accuracy may mislead the performance of the classifier. Some evaluation methods are selected to assess the class-imbalanced classifier.

(11) $$Accuracy=\frac{TP+TN}{TP+TN+FP+FN}\;$$

For class-imbalanced fall detection tasks, the sensitivity, specificity, F-score and receiver operating characteristic (ROC) can effectively reflect the ability of the classifier to distinguish falls from a large number of ADL events. Sensitivity (Equation (12)) is also called precision, which measures the proportion of positives that are correctly identified. Specificity (Equation (13)) measures the proportion of negatives that are correctly identified.

(12) $$Sensitivity=\frac{TP}{TP+FN}\;$$

(13) $$Specificity=\frac{TN}{TN+FP}$$

The F-score (Equation (14)) is a robust method that measures the discrimination of sensitivity and specificity. Generally, sensitivity and specificity are often mutually restricted in large-scale datasets. A trade-off is often needed depending on the specific situation. β is the parameter that mediates the weight of sensitivity and specificity. When β=1, both the sensitivity and specificity are of equal weight. If β<1, the specificity is more important than sensitivity. The sensitivity will be assigned higher weight when β>1. In fall detection, the specificity reflects whether all fall signals in the data are detected and it deserves a higher weight. In this research, β is set to 0.5.

(14) $$F-Score=\left(1+\beta^{2}\right)\times \frac{Sensitivity\times Specificity\;}{\beta^{2}\times \left(Sensitivity+Specificity\;\right)}$$

The receiver operating characteristic (ROC) is a plot of sensitivity (often called the true positive rate) versus 1-specificity (often called the false positive rate) that offers a summary of sensitivity and specificity across a range of cut points for a continuous predictor. It is an evaluation of the binary classification problem, and the curve is not affected by the class imbalance in the dataset. The area under the curve (AUC) is defined as the area under the ROC curve. The AUC value is equivalent to the probability that a randomly chosen positive example is ranked higher than a randomly chosen negative example. AUC ranges from 0 to 1, and 1 indicates a perfect classifier that can distinguish all data correctly.

### 4.4. Experimental Results

The architecture proposed in Section 3 was implemented in 2.4.0 TensorFlow [45] using the Keras API. The training and testing experiments were performed on an GeForce RTX 3090 GPU (Nvidia, CA, USA).

In order to thoroughly evaluate the performance of the proposed model on the fall detection problem, the five latest deep learning models were selected for comparison. Table 3 illustrates the architecture of the networks: CNN [46], LSTM [47], DeepSense [48], ResNet [33]. These models have been proposed to solve time-series classification tasks including fall detection problems and have achieved great success. First of all, the preprocessed data were randomly divided into train, validation and test sets in the same ratio. The ratio of train, validation and test was 6:2:2. Each model was trained for a maximum of 30 epochs, and the learning rate was set to 0.001. Table 4 shows how the models performed on the SisFall dataset.

**Figure 8:**
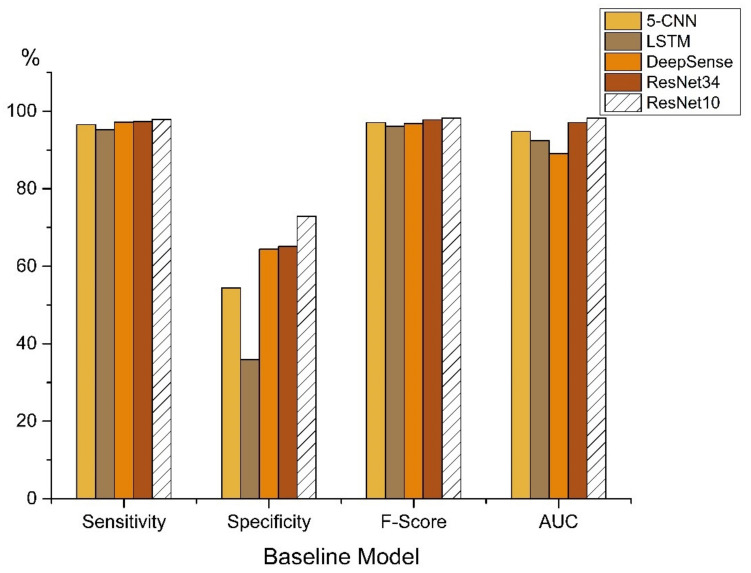
Results of comparison of different deep learning baseline models.

In Table 4 and Figure 8, the results of these models are summarized. As shown in the table, the ResNet model performs significantly better on this dataset when compared to the other models. As very deep networks harm the generalization performance of the classifier, ResNet34 has poorer performance than ResNet10. In the last five baseline models, ResNet10 performs the best among all evaluation methods. It reaches an F-score of 98.25% and an AUC of 98.27%. In particular, the ResNet10 model achieves specificity of 72.89%, which is 36.98% higher than that of the LSTM model. These baseline models can successfully find the features within given sensor data, but the specificity is not high due to the bias of the majority group on the class-imbalanced data.

In order to evaluate the performance of the proposed approach, CDL-Fall, we conducted experiments to compare its performance with the state-of-the-art algorithms, including Class-Weight [30], SMOTE [26], SMOTE+Threshold [31] and Focal Loss [28]. These algorithms have already been proven to work well in class-imbalanced tasks, such as computer vision [29], disease detection [49], fraud detection [50] and others [51].

**Figure 9:**
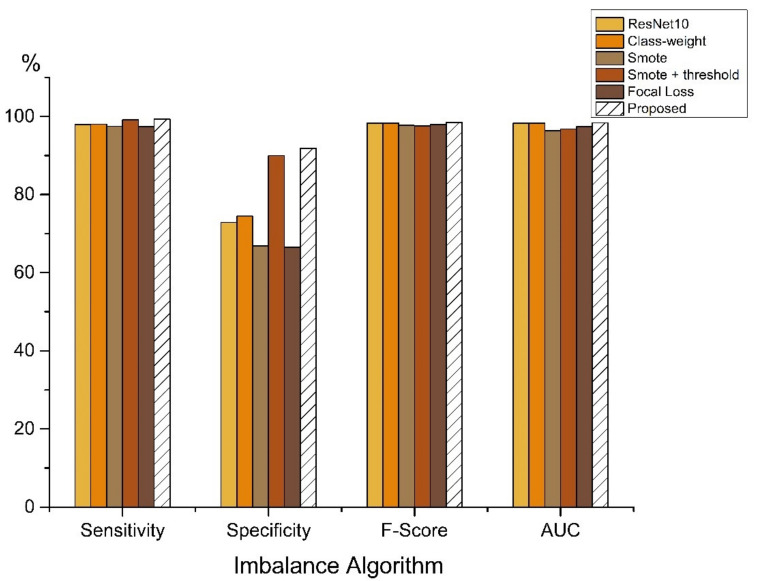
Results of comparison of imbalance algorithm of ResNet model.

As shown in Table 5 and Figure 9, the five types of imbalance algorithm optimization methods on the baseline model can successfully identify fall events, and the proposed method achieves the best scores among all evaluation methods. Compared to the baseline model, the proposed method improves the specificity from 72.89% to 91.86%. The worst model was the SMOTE model, with only 66.88%. The F-score and the AUC are also higher than those of the baseline model.

## 5. Conclusions

Fall detection in real life can be treated as an anomaly detection problem due to the class-imbalanced data. A fall detection classifier trained on ideal class-balanced data may lead to missing fall data. The purpose of the work was to develop an algorithm that could automatically extract features from raw class-imbalanced training data.

In this paper, the CDL-Fall algorithm is proposed. The model is based on the ResNet model and demonstrates improved performance on the SisFall dataset. According to the evaluation results, compared with the-state-of-the-art fall detection algorithms, CDL-Fall can achieve the highest score in multiple evaluation methods, with sensitivity of 99.33%, specificity of 91.86%, an F-score of 98.44% and an AUC of 98.35%. In particular, regarding the specificity, it is greatly improved from 72.89% to 91.86%. It can effectively reduce the missed detection of fall events. The results presented in this research confirm the effectiveness of using CDL-Fall in the presence of class-imbalanced data.

## Figures and Tables

**Table 1:** Comparison of imbalance ratio in previous studies.

Authors	Num of Samples	Num of ADL Samples	Num of Fall Samples	Imbalance Ratio
Bourke et al. [13]	480	240	240	1.00
Casilari et al. [19]	530	371	159	2.33
Li et al. [20]	350	225	125	1.8
Yu et al. [22]	585	385	200	1.925
Martinez-Villaseor et al. [23]	559	304	255	1.19
Wang et al. [24]	3580	1790	1790	1.00
Waheed et al. [25] *	770	395	375	1.05
	8250	4530	3720	1.22

* Two different datasets are used in this paper.

**Table 2:** The details of publicly available fall datasets.

Dataset	Num of Type of ADLs/Falls	Num of Samples (ADLs/Falls)	Type of Sensors *
DLR [34]	15/1	1017 (961/56)	A, G, M
MobiAct [35]	9/4	2526 (1879/647)	A, G, O
TST Fall detection [36]	4/4	264 (132/132)	A
tFall [37]	7/8	10,909 (9883/1026)	A
UR Fall Detection [38]	5/4	70 (40/30)	A
Cogent Labs [39]	8/6	1968 (1520/448)	A, G
Gravity Project [40]	7/12	117 (45/72)	A
Graz [41]	10/4	2460 (2240/220)	A, O
UMAFall [19]	8/3	531 (322/209)	A, G, M
SisFall [42]	19/15	4505 (2707/1798)	A, A, G
UniMiB SHAR [43]	9/8	7013 (5314/1699)	A
UP-Fall [44]	6/5	559 (304/255)	A, G

* A: Accelerometer, G: Gyroscope, M: Magnetometer, O: Orientation Measurements.

**Table 3:** The architecture of deep learning baseline models.

Model	Architecture
5-CNN	Input + 5 × (Convolution + ReLU) + Softmax
LSTM	Input + 2 × (LSTM + Dropout) + Softmax
DeepSense	3 × (Input + Convolution) + Concatenate + 2 × LSTM + Softmax
ResNet34	Input + (3 + 4 + 6 + 3) × (Convolution + ReLU) + Softmax
ResNet10	Input + (1 + 1 + 1 + 1) × (Convolution + ReLU) + Softmax

**Table 4:** Comparison of different deep learning baseline models.

Model	Sensitivity (%)	Specificity (%)	F-Score (%)	AUC (%)
5-CNN	96.54	54.41	97.10	94.81
LSTM	95.21	35.91	96.09	92.41
DeepSense	97.17	64.37	96.84	89.03
ResNet34	97.33	65.08	97.79	97.06
ResNet10	97.91	72.89	98.25	98.27

**Table 5:** Comparison of imbalance algorithm of ResNet model.

Method	Sensitivity (%)	Specificity (%)	F-Score (%)	AUC (%)
Baseline (ResNet10)	97.91	72.89	98.25	98.27
Class-Weight	98.03	74.49	98.31	98.31
SMOTE	97.49	66.88	97.70	96.39
SMOTE + Threshold	99.17	89.98	97.56	96.80
Focal Loss	97.44	66.49	97.90	97.39
Proposed	99.33	91.86	98.44	98.35

## Data Availability

The experiments were performed on a publicly available dataset. The source of the utilized dataset is available in [42].
